# Effectiveness and efficiency of primary care based case management for chronic diseases: rationale and design of a systematic review and meta-analysis of randomized and non-randomized trials [CRD32009100316]

**DOI:** 10.1186/1472-6963-10-112

**Published:** 2010-05-07

**Authors:** Tobias Freund, Felizitas Kayling, Antje Miksch, Joachim Szecsenyi, Michel Wensing

**Affiliations:** 1 Competence Centre of General Practice, University Hospital Heidelberg, Department of General Practice and Health Services Research, Voßstrasse 2, 69115 Heidelberg, Germany; 2 Radboud University Nijmegen Medical Centre, Scientific Institute for Quality of Healthcare, P.O. Box 9101, 6500HB Nijmegen, Netherlands

## Abstract

**Background:**

Case management is an important component of structured and evidence-based primary care for chronically ill patients. Its effectiveness and efficiency has been evaluated in numerous clinical trials. This protocol describes aims and methods of a systematic review of research on the effectiveness and efficiency of case management in primary care.

**Methods/Design:**

According to this protocol Medline, Embase, CINAHL, PsychInfo, the Cochrane Central Register of Controlled trials, DARE, NHS EED, Science Citation Index, The Royal College of Nursing Database, Dissertation Abstracts, registers of clinical trials and the reference lists of retrieved articles will be searched to identify reports on randomized and non-randomized controlled trials of case management interventions in a primary care setting without limitations on language or publication date. We will further ask experts in the field to avoid missing relevant evidence. Study inclusion and data extraction will be performed independently by two reviewers. After assessing risk of bias according to predefined standards, included studies will be described qualitatively. Subgroup analyses are planned for different chronic diseases and intervention strategies. If appropriate, a quantitative synthesis of data will be performed to provide conclusive evidence about the effectiveness and efficiency of primary care based case management in chronic care.

**Review registration:**

Centre for Reviews and Dissemination (University of York): CRD32009100316.

## Background

The concept of case management is thought to be an effective and efficient approach to manage patients with chronic illness and complex health care needs [1]. Case management can implement key elements of the chronic care model, such as improved continuity of care by redesigning the delivery system and enhancing patients' self-management skills [2]. It can also contribute to better implementation of evidence-based recommendations for diagnostic procedures, pharmaceutical treatment, life style counselling and monitoring of patients.

Case management has been implemented in a range of clinical settings [3] but most chronically ill patients receive most of their medical care in primary care settings, at least in countries with a strong primary care system. Therefore this seems to be the most obvious setting for case management programs. A previous review on case management in primary care concluded that interventions supervised by generalists were not effective in reducing health resource use [4]. Since then the body of evidence has vastly expanded so that an update of this reviews was required.

**Figure 1:**
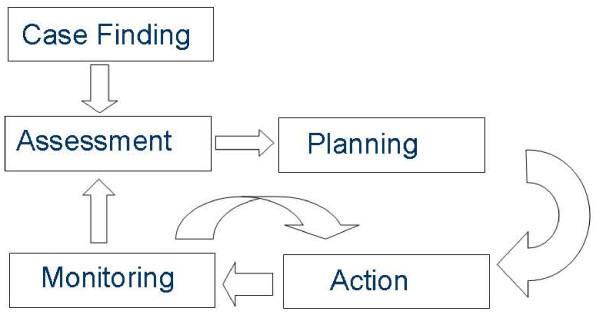
**The principle case management process**. The figure shows the principle process of varying case management approaches. It consists of "case finding" and "individualized assessment" followed by "planning" different "actions" which are "monitored" and/or re-assessed with implications on future plans and actions.

Case management is rather a generic concept than a clearly defined intervention strategy [5]. But conclusive answers about the effects of complex interventions like case management programs demand precise definitions for the inclusion of evidence [6]. Therefore we aim to base the selection of studies on a basic principle of case management which can be found in various concepts [7-9] (figure 1). The principle case management process emphasises on highly intensive individualized care contrasting *case *management to *disease *management programs [10]. Case Management starts with identifying cases in need for intensified management. As a next common step needs assessment and individualized planning are implemented in different concepts. Monitoring and/or re-assessment of the various actions which could be planned to manage the cases are more or less explicit elements of various case management approaches. All components of this process can be undertaken in different settings. In the context of our interest on primary care based case management we defined the involvement of a primary care physician (either general internist, general practitioner, family physician) in planning the management of individual cases as being essential.

Using a typology to guide (sub)classification of interventions could further improve the quality of systematic reviews on complex interventions [6]. The typology proposed by the Cochrane Consumer and Communication Review Group may be useful to classify different primary care based case management interventions from the perspective of the interacting "cases" and "managers" [11]. This may help answering the question "why" some case management interventions in primary care work and others do not.

We aim to review existing evidence on chronic diseases that can be effectively and efficiently cared for by implementing case management in primary care. We will further search for key components of case management which due to success or failure of these programs.

## Methods/Design

This systematic review is performed according to standards derived from the PRISMA Statement [12].

### Eligibility criteria

#### Types of studies

We include randomised and non-randomised controlled trials studying the effects and/or economic implications of case management interventions compared to routine care. No language, publication date or publication status restrictions will be made. Study protocols will also be included and we aim to contact authors to provide details about ongoing publications.

#### Types of participants

Studies on adult participants (18 years and above) suffering from at least one chronic condition will be included in the review. Reports on interventions of palliative care, cancer screening, primary prevention, and treatment of drug or substance abuse are excluded from this review.

#### Type of intervention

Trials comparing case management interventions in which primary care physicians (alone or in collaboration with specialists) were involved in planning of the management strategy for individual cases will be included in this review. Case management is defined as consisting of all elements of the case management process as described above (figure 1). Monitoring in this context is defined as a periodic assessment (at least once in six months) which can result in a change of managing the case. We aim to contact authors in the case of ambiguities about key components due to lack of reporting in publications. We will include studies comparing case management interventions with routine management (usual care, attention control). The content of "usual care" will be carefully described in the review.

### Information sources

We will search Medline via OVID (1950- 2009), the Cochrane Central Register of Controlled trials (2009), DARE, Embase (1980 to 2009), CINAHL (1982-2009), NHS EED, PsychInfo (1887- 2009), Science Citation Index (1987- 2009), Royal College of Nursing database, Dissertation abstracts (1861-2009) and Registers for clinical trials. The search is not limited to language or publication date. Searches will be designed and conducted by TF, AM and MW and assisted by librarians. Additionally we aim to search reviews relevant for the topic to retrieve further studies of interest. The reference lists of retrieved reports will be screened for potentially relevant studies. We will ask experts in the field for support to avoid missing relevant studies.

### Search

We use a highly sensitive search filter for randomized controlled trials [13] and add "usual care" in the filter as this is a common term used for describing the control group of case management trials. We will identify studies of relevance using the MeSH terms: primary health care.exp and patient care planning.exp. A search in titles and abstracts will be performed using a combination of free text terms: case manage$.tw, care manage$.tw, disease manage$.tw, integrated care.tw, family medicine.tw, family practice.tw, general prac$.tw, primary care.tw.

### Study selection

Eligibility assessment will be performed independently by two reviewers (TF, FK). Studies will be selected unblinded according to a standardized procedure. Abstracts with incongruent assessment results will be included in full text screening without further consensus discussion. Disagreements during the full text-based study selection process will be discussed and resolved by consensus.

### Data collection

Two investigators will extract data from each study independently. We use a standardized protocol and reporting form to extract trial characteristics, patient data, outcomes and study quality. The data extraction instrument will be piloted with 5 study reports at minimum which will be included in this review. We aim to refine the instrument after the pilot test. Authors of published study protocols will be asked to provide information about first results and planned publications if results have not already been published.

We will extract data from doubled reports of studies according to the following algorithm [1. review of study protocol, 2. review of the major publication i.e. published in a "high-impact" journal, report of major outcome, 3. review of all other publications with quantitative data, 3. contact to authors in the case of inconsistencies within reports]. All data of a single study will be displayed comprehensively in the review even if reported in different publications.

### Data items

We will design a data extraction form containing the following information: (1) characteristics of participants (age, gender, disease, disease stage and severity, method of diagnosis, drop-outs), and the trial's inclusion and exclusion criteria; (2) type of intervention (case finding, assessment, planning, intervention, monitoring, re-assessment; versus usual care or versus attention control); (3) type of outcome measure (including symptom level, quality of life score [using a validated tool], quality of care score [validated tool], adherence [validated tool], mortality, functional status, clinical parameters, surrogate parameters [e.g. HbA1c], admission rate, days of hospitalisation, outpatient care visits, emergency department visits, direct or indirect costs, length of follow-up, unintended effects of treatment).

### Risk of bias in individual studies

To assess the validity of included studies pairs of two reviewers will rate risk of bias according to predefined standards using the Cochrane Collaboration's tool for assessing risk of bias [13]. This tool has been validated but a detailed checklist is needed to use it appropriately [14]. Using the risk of bias tool adapted by the EPOC Group [15] we created a checklist which we would like to publish *a priori *as recently suggested [16] (see additional file 1: Risk of bias assessment checklist).

### Planned methods for analysis

As a first step, we will perform qualitative synthesis of included studies using summary of findings tables. Intervention strategies will carefully be described with emphasis on the interactions between case management teams and patients using a Cochrane typology [11]. The content of usual care, intensity of case management interventions and training of case managers will further be used to compare included studies. Heterogeneity is a common problem of data synthesis from evaluations of complex interventions [6]. We will perform subgroup analyses regarding the following domains: different chronic conditions, training of case managers and intensity of the intervention. Heterogeneity tests will be calculated within subgroups. We aim to synthesize data quantitatively if adequate to the findings. Any meta-analysis will be carried out using a random effects model. Single effect sizes (standardised mean differences) may be more appropriate to report quantitative synthesis in order to compare different outcome measures. If possible, sensitivity analyses are planned to simulate different levels of involvement of primary care physicians in planning. Sensitivity and subgroup analysis will also be undertaken for studies with largely different risk of bias.

## Abbreviations

EPOC: Effective Practice and Organization of Care Group; PRISMA: Preferred Reporting Items for Systematic Reviews and Meta-Analyses; CINAHL: Cumulative Index to Nursing and Allied Health; NHS EED: NHS Economic Evaluation Database; DARE: Database of Abstracts of Reviews of Effects.

## Competing interests

The authors declare that they have no competing interests.

## Authors' contributions

TF leads the review and developed the study protocol. TF, FK, AM and MW contributed substantially to the design of the search strategy and abstraction of data. TF wrote the manuscript. AM, JS and MW critically revised it. All authors read and approved the final manuscript.

## Pre-publication history

The pre-publication history for this paper can be accessed here:

http://www.biomedcentral.com/1472-6963/10/112/prepub

## Supplementary Material

Additional file 1 **Risk of bias assessment checklist**. This file contains a detailed checklist for the assessment of risk which will be used in this review. It is an adapted version of the tool provided by the Cochrane EPOC Group. Click here for file

## References

[B1] Bodenheimer T Berry-Millett R Care Management of Patients with Complex Health Care Needs 2009 Robert Wood Johnson Foundation http://www.policysynthesis.org 22052205

[B2] Wagner EH Austin BT Davis C Hindmarsh M Schaefer J Bonomi A Improving chronic illness care: translating evidence into action Health Aff (Millwood) 2001 20 64 78 10.1377/hlthaff.20.6.64 11816692

[B3] Lee DT Mackenzie AE Dudley-Brown S Chin TM Case management: a review of the definitions and practices J Adv Nurs 1998 27 933 939 10.1046/j.1365-2648.1998.t01-1-00566.x 9637319

[B4] Ferguson JA Weinberger M Case management programs in primary care J Gen Intern Med 1998 13 123 126 10.1046/j.1525-1497.1998.00029.x 9502373 PMC1496915

[B5] Zwarenstein M Reeves S Strauss SE Pinfold P Goldman J Case management: effects on professional practice and health care outcomes (Protocol) Cochrane Database Syst Rev 2009 1 CD002797 doi:10.1002/14651858.CD002797.

[B6] Shepperd S Lewin S Straus S Clarke M Eccles MP Fitzpatrick R Wong G Sheikh A Can we systematically review studies that evaluate complex interventions? PLoS Med 2009 6 e1000086 10.1371/journal.pmed.1000086 19668360 PMC2717209

[B7] Bergen A Case management in community care: concepts, practices and implications for nursing J Adv Nurs 1992 17 1106 1113 10.1111/j.1365-2648.1992.tb02045.x 1401551

[B8] Gensichen J Beyer M Kuver C Wang H Gerlach FM Case management for patients with congestive heart failure under ambulatory care--a critical review Z Arztl Fortbild Qualitatssich 2004 98 143 154 15106496

[B9] The Case Management Society of America http://www.cmsa.org/

[B10] Krumholz HM Currie PM Riegel B Phillips CO Peterson ED Smith R Yancy CW Faxon DP A taxonomy for disease management: a scientific statement from the American Heart Association Disease Management Taxonomy Writing Group Circulation 2006 114 1432 45 10.1161/CIRCULATIONAHA.106.177322 16952985

[B11] The Cochrane Consumer and Communication Review Group http://www.latrobe.edu.au/chcp/assets/downloads/TopicList.pdf

[B12] Liberati A Altman DG Tetzlaff J Mulrow C Gotzsche PC Ioannidis JP Clarke M Devereaux PJ Kleijnen J Moher D The PRISMA statement for reporting systematic reviews and meta-analyses of studies that evaluate health care interventions: explanation and elaboration J Clin Epidemiol 2009 62 e1 34 10.1016/j.jclinepi.2009.06.006 19631507

[B13] The Cochrane Handbook for systematic reviews of interventions http://www.cochrane.org/resources/handbook/index.htm

[B14] Hartling L Ospina M Liang Y Dryden DM Hooton N Krebs Seida J Klassen TP Risk of bias versus quality assessment of randomised controlled trials: cross sectional study BMJ 2009 339 b4012 doi: 10.1136/bmj.b4012. 10.1136/bmj.b4012 19841007 PMC2764034

[B15] Cochrane Effective Practice and Organisation of Care Group http://www.epoc.cochrane.org/en/index.html

[B16] Freund T Hartling L, Ospina M, Liang Y, Dryden DM, Hooton N, Krebs Seida J, Klassen TP Improving the quality of quality assessment in systematic reviews Risk of bias versus quality assessment of randomised controlled trials: cross sectional study. BMJ 2009 339 b4012 10.1136/bmj.b4012 PMC2764034 19841007

