# Supplementary material for: Effectiveness and efficiency of primary care based case management for chronic diseases: rationale and design of a systematic review and meta-analysis of randomized and non-randomized trials [CRD32009100316]
Source: BMC Health Serv Res. 2010 May 7;10:112. doi: 10.1186/1472-6963-10-112 (PMC2907755; doi:10.1186/1472-6963-10-112)
Supplement: Additional file 1 — Risk of bias assessment checklist. This file contains a detailed checklist for the assessment of risk which will be used in this review. It is an adapted version of the tool provided by the Cochrane EPOC Group. [file 1472-6963-10-112-S1.PDF]

Risk of bias	Description	Details on reviewer judgements		
		„Yes“	„No“	„Unclear“
**Adequate sequence generation?**	Methods used to generate allocation sequence in order to provide comparable groups	- random number table<br>- computer random number generator<br>- coin tossing<br>- shuffling cards or envelopes<br>-throwing dice<br>-drawing lots	- nonrandomized trial<br>-by odd or even date of birth<br>- some rule based on date of birth or admission<br>- based on hospital or clinic record number<br>- by judgment of clinician<br>- participants choice<br>-based on test results<br>- based on availability of the intervention	- insufficient information reported
**Allocation concealment?**	Methods used to conceal allocation: Was it possible to foresee group allocation in advance of or during enrolment?	- allocation of all clusters (institutions, teams) or professionals at the beginning of the study<br>- central allocation of patients<br>- on-site computerized allocation of patients<br>- sequentially numbered, opaque, sealed envelopes	- open random allocation schedule (e.g. list of random numbers)<br>- open or non-opaque envelopes<br>- alternation or rotation<br>- date of birth<br>- case record number	- insufficient information reported
**Blinded outcome assessment?**	Assessor of primary outcomes blinded to allocation of participants	- Blinded assessor of subjective primary outcome (e.g. quality of life, patients´ satisfaction)<br>- objective outcome (e.g. hospital admissions)	- subjective outcomes not blindly assessed	- insufficient information reported
**Incomplete outcome data addressed?**	Completeness of outcome data for main outcomes. Report on reasons for exclusion, attrition.	- No missing data<br>- missing data balanced in numbers in compared groups<br>- proportion of missing data compared to measured effects unlikely to be clinically relevant<br>- appropriate imputation methods	- imbalance in numbers or reasons across groups<br>- missing outcome data likely to be clinically relevant compared to effect size<br>- “as-treated” analysis and large difference between intervention received and allocated to intervention<br>- inadequate simple imputation	- insufficient information on numbers/reasons for missing outcome data

**Free of selective reporting?**	Relevant outcomes measured but not reported	- all relevant outcomes from the method section report in result part<br>- study protocol available and all primary and secondary outcomes of interest for the review are reported	- not all of the pre-specified outcomes have been reported<br>- outcomes reported were not be pre-specified (unless reason explicitly reported)<br>- study report fails to include results from key outcome that would be expected from study design<br>- using of measurements, analysis or subsets of data not pre-specified	- no study protocol available<br>- insufficient information to rate this topic
**Baseline measurements?**	Baseline measurement of outcomes and characteristics of participants	- baseline characteristics of providers and patients reported and similar<br>- outcome at baseline reported and similar or analysis adjusted for imbalance	- important imbalances in outcome at baseline and analysis not adjusted<br>- no report on baseline characteristics<br>- important difference in baseline characteristics in groups (e.g. due to recruitment bias)	- no measurement of outcome at baseline<br>- data baseline characteristics insufficiently reported
**Free of contamination bias?**	Risk of control group receiving the intervention	- allocation at community, institution, practice level	- randomization at patient level	- professionals allocated within the same institution and communication may occur between them
**Valid instruments?**	Use of valid instruments for outcome measurement	- instruments referenced to validation studies<br>- self-designed instruments piloted with information given about the results of the pilot	- self-designed instruments without piloting/reports on validation	- insufficient information reported about instruments used in the study
**Free of other risk of bias?**			- study interrupted due to data-dependent process<br>- claimed to have been fraudulent<br>- design effect of cluster randomized trials not reported with analysis	- identified problem but not clear evidence of being bias
